# Chromatin regulators, phenotypic robustness, and autism risk

**DOI:** 10.3389/fgene.2014.00081

**Published:** 2014-04-10

**Authors:** Reut Suliman, Eyal Ben-David, Sagiv Shifman

**Affiliations:** Department of Genetics, The Institute of Life Sciences, The Hebrew University of JerusalemJerusalem, Israel

**Keywords:** autism spectrum disorder, *de novo* mutation, chromatin regulators, phenotypic robustness, common genetic variants

## Abstract

Though extensively characterized clinically, the causes of autism spectrum disorder (ASD) remain a mystery. ASD is known to have a strong genetic basis, but it is genetically very heterogeneous. Recent studies have estimated that *de novo* disruptive mutations in hundreds of genes may contribute to ASD. However, it is unclear how it is possible for mutations in so many different genes to contribute to ASD. Recent findings suggest that many of the mutations disrupt genes involved in transcription regulation that are expressed prenatally in the developing brain. *De novo* disruptive mutations are also more frequent in girls with ASD, despite the fact that ASD is more prevalent in boys. In this paper, we hypothesize that loss of robustness may contribute to ASD. Loss of phenotypic robustness may be caused by mutations that disrupt capacitors that operate in the developing brain. This may lead to the release of cryptic genetic variation that contributes to ASD. Reduced robustness is consistent with the observed variability in expressivity and incomplete penetrance. It is also consistent with the hypothesis that the development of the female brain is more robust, and it may explain the higher rate and severity of disruptive *de novo* mutations in girls with ASD.

## Introduction

The genetic architecture of ASD is very complex and hard to dissect. Recently, there have been several breakthroughs in our understanding of this heterogeneous disease, including increased appreciation of the importance of *de novo* mutations (Sebat et al., 2007). We now know that ASD can be triggered by different types of genetic variations in many different genes, a phenomenon termed non-allelic genetic heterogeneity. It is estimated that mutations that are likely to disrupt gene function (such as nonsense, splice site and frameshift mutations) in hundreds of different genes may contribute to ASD (Iossifov et al., 2012; Neale et al., 2012). The involvement of rare mutations was clear from the finding that ASD is associated with genetic syndromes that show Mendelian inheritance (Cook et al., 1997; Manning et al., 2004; Splawski et al., 2004; Abrahams and Geschwind, 2008). In addition, rare and *de novo* large chromosomal rearrangements are also found more frequently in ASD, currently estimated to account for 2–7% of cases (Marshall et al., 2008; Shen et al., 2010).

Recently, with the advent of molecular tools that enable to study the genome in high resolution and unbiased way, *de novo* variations, both copy number variations (CNVs) and single nucleotide variations (SNVs), have been found to be associated with ASD, currently estimated to account for 6 and 10% of the cases, respectively (Sebat et al., 2007; Pinto et al., 2010; Sanders et al., 2011; Iossifov et al., 2012; Neale et al., 2012; O'Roak et al., 2012b; Sanders et al., 2012). Based on the rate of *de novo* SNVs it has been estimated that hundreds of genes can lead to ASD when disrupted (Iossifov et al., 2012; Neale et al., 2012; O'Roak et al., 2012b; Sanders et al., 2012). Consequently, each gene could account for only a small proportion of cases.

## Molecular mechanisms affected by *rare de novo* mutations

The current view of the genetic architecture of ASD is that it is similar to other complex diseases. ASD risk is attributed to both rare genetic variants and combinations of common variants that act together with environmental risk factors (Huguet et al., 2013). It is assumed that people with ASD have a set of genetic variants that predispose them to abnormal development of specialized brain structures involved in social information processing (the “social brain”). Thus, it is puzzling that there is no unifying pathophysiology in ASD, in addition to the findings of mutations in many different genes, involved in different functions.

A possible solution to this puzzle would be if shared mechanisms between some of those genes could be identified. First attempts to identify such mechanisms focused on genes within rare copy-number variations (*de novo* or inherited) (Pinto et al., 2010; Gai et al., 2011; Gilman et al., 2011). One such effort found enrichment in rare deletions for genes involved in neuronal development and function, as well as for GTPase/Ras signaling (Pinto et al., 2010). Other studies found enrichment in CNVs for genes involved in synaptic transmission (Gai et al., 2011) and synaptogenesis (Gilman et al., 2011). It is important to note, however, that enrichment studies in CNVs have several limitations. First, the power of enrichment analyses based on CNVs is limited by their large size, which in many cases encompasses a large number of genes. Furthermore, in such analyses gene size may also be an important confounding factor, especially when dealing with disorders of the brain (Raychaudhuri et al., 2010). Another approach was to study genes with single base mutations; including mutations that lead to ASD associated genetic syndromes (Sakai et al., 2011; Ben-David and Shifman, 2012). However, many of these genes have been discovered in studies which were at least partly hypothesis driven, and used previous knowledge to identify the causal gene in large inherited chromosomal aberrations or segregating loci in families with ASD.

The advent of next generation sequencing, and with it the recent sequencing of four large ASD cohorts of families for *de novo* exonic mutations (Iossifov et al., 2012; Neale et al., 2012; O'Roak et al., 2012b; Sanders et al., 2012), has provided an unprecedented sample of *de novo* disruptions which have been discovered in an unbiased genome-wide manner, each affecting a specific gene. When combining the mutations found in these cohorts and performing a functional enrichment analysis, an enrichment was found for genes involved in transcription regulation, and in particular chromatin regulators that control chromatin structure and function (Table 1) (Ben-David and Shifman, 2013). This enrichment is apparent when concentrating on genes with mutations highly likely to be functional—nonsense, frame shift or splice site mutations (Ben-David and Shifman, 2013). When looking at the temporal expression pattern of these genes during brain development, they were found to be strongly expressed prenatally, with much lower expression after birth (Ben-David and Shifman, 2013). The specificity of the enrichment of transcription regulators to ASD was demonstrated by comparing the distribution of mutations between cases and a large set of controls, and to unaffected siblings. Furthermore, by comparing the distribution of disruptive vs. silent mutations in the same subjects it was shown that the enrichment of chromatin regulators is unlikely to be a result of detection or mutation bias (Ben-David and Shifman, 2013). Still it is not clear how disruptive mutations in chromatin regulators and other genes involved in regulation of transcription link to ASD. We suggest below that mutation in chromatin regulators causes loss of the robustness of brain development that together with other ASD specific risk factors may lead to ASD.

**Table 1:** Chromatin regulators associated with autism spectrum disorders^*^.

**Gene symbol**	**Chromosomal location**	**Support for autism**	**Evidence of support**
ADNP	20q13.13	Rare single gene mutations	O'Roak et al., 2012a,b
ARID1B	6q25.1	Rare single gene mutations	Halgren et al., 2011; Nord et al., 2011
CHD7	8q12.2	Syndromic—CHARGE syndrome	Vissers et al., 2004; O'Roak et al., 2012b
CHD8	14q11.2	Rare single gene mutations	O'Roak et al., 2012a,b; Talkowski et al., 2012
CREBBP	16p13.3	Syndromic—Rubinstein-Taybi syndrome (RTS)	Petrij et al., 1995
HDAC4	2q37.3	Syndromic—brachydactyly mental retardation syndrome	Williams et al., 2010a
MBD5	2q23.1	Rare single gene mutations	Wagenstaller et al., 2007; Jaillard et al., 2009; Van Bon et al., 2010; Williams et al., 2010b; Talkowski et al., 2011, 2012; Hodge et al., 2013
MECP2	Xq28	Syndromic—Rett syndrome	Amir et al., 1999
NSD1	5q35	Syndromic—Sotos syndrome	Kurotaki et al., 2002
POGZ	1q21.3	Rare single gene mutations	Iossifov et al., 2012; Neale et al., 2012
SETD2	3p21.31	Rare single gene mutations	O'Roak et al., 2012a,b
SUV420H1	11q13.2	Rare single gene mutations	Iossifov et al., 2012; Sanders et al., 2012

* *Adapted from SFARI gene database on July 16th 2013, chromatin regulators were taken if they were either associated with syndromic ASD, or had rare mutations in at least two different individuals*.

## Decreased robustness in ASD

Phenotypic robustness is the insensitivity of the phenotype to genetic or environmental perturbations. An equivalent concept is developmental stability, which is defined as the ability of the individual to produce a robust phenotype even if faced with genetic and environmental perturbations during development. A further related term is canalization, which refers to the selection during evolution for more stable phenotypes, or in other words, adaptive robustness (Gibson and Wagner, 2000). In the following, we will use the term phenotypic robustness without the distinction between adaptive and intrinsic forms of robustness (Gibson and Wagner, 2000). Loss of phenotypic robustness has been recently proposed to explain the missing heritability in complex disease (Gibson, 2009; McGrath et al., 2011; Queitsch et al., 2012). Based on this proposal, the degree of robustness varies between individuals and it influences the probability of developing a disease or disorder. It was also argued that findings in ASD and schizophrenia may be consistent with decreased robustness, a phenomenon termed decanalization (Woolf, 1997; McGrath et al., 2011; Queitsch et al., 2012). Furthermore, it was speculated that brain structures (such as the neocortex) that have undergone profound evolution in the recent lineage leading to humans, may be more vulnerable to loss of robustness because there has been insufficient time to evolve sufficient buffering capacity (McGrath et al., 2011). The brain may be also more vulnerable than other organs, because it is a complex organ that develops later in life and mainly composed of terminally differentiated cells. In the first years of life, the brain may be particularly sensitive to reduced robustness because the social development depends on signals from the environment.

The development of the brain is generally robust, so it may seem surprising that *de novo* mutations in the heterozygote state contribute to ASD and other neurodevelopmental disorders. Many genes can be inhibited in mice without producing a phenotype. Moreover, heterozygous knockout (KO) mice rarely show a clear phenotype; In fact, in many experiments heterozygous KO mice are often used as a control for homozygote KO mice. While neurodevelopmental systems have evolved to be robust, they may be vulnerable to perturbations in a specific subset of genes, termed phenotypic capacitors (Rutherford and Lindquist, 1998; Levy and Siegal, 2008). Phenotypic capacitors are genes that buffer against perturbations and therefore contribute to the robustness of the phenotype. So, phenotypic capacitors, when operating normally, may prevent the development of disorders like ASD even in individuals carrying or exposed to genetic and environmental risk factors.

The most studied capacitor is Hsp90 (Rutherford and Lindquist, 1998). Hsp90 was termed “genetic capacitor” because the reduction in its activity increased phenotypic variation. It was first suggested that its chaperone activity, that facilitate the correct folding of proteins with destabilizing mutations, is how Hsp90 can buffer mutations (Tokuriki and Tawfik, 2009). However, more recent work has suggested that Hsp90 prevents phenotypic variation by suppressing transposon activity (Specchia et al., 2010). Besides Hsp90, yeast and worm studies strongly implicate chromatin regulators to be phenotypic capacitors (Lehner et al., 2006; Levy and Siegal, 2008; Tirosh et al., 2010). In yeast, more than 300 genes are known to be important for robustness. Very similar to the type of genes implicated in ASD, the phenotypic capacitors in yeast tend to be highly connected regulatory genes, many of which are involved in transcription and chromatin regulation (Levy and Siegal, 2008). A major source of variation during development is stochastic fluctuations in gene expression (McAdams and Arkin, 1997). Gene expression shows significant variation, even between genetically identical cells (Lehner, 2008). In this setting, chromatin regulators and other transcription factors are likely critical to the ability of a cell to buffer the fluctuations in gene expression against cryptic genetic variations (Tirosh et al., 2010). Cryptic genetic variations are hidden genetic variations that do not affect the trait in a given genetic background or environmental condition, but may be expressed after environmental, genetic, or epigenetic perturbations (Gibson and Dworkin, 2004).

The chromatin regulators that were found to be disrupted by *de novo* mutations in ASD may be involved in multiple cellular processes, such as regulation of transcription, cell cycle regulation, genomic stability, and DNA damage repair. Still it is not clear how mutations in chromatin regulators lead to ASD. While collectively *de novo* mutations in chromatin regulators account for a small proportion of cases, understanding how they operate may shed light on the biological mechanisms of ASD. Following the models of loss of robustness, we suggest that mutations that affect chromatin regulators may lead to ASD because they are “global regulators,” namely they are genes that are placed at the very top of a regulatory hierarchy. Chromatin regulators interact with many genes and pathways, and so perturbation in them can affect multiple target genes simultaneously. Furthermore, we propose that these genes may also act as phenotypic capacitors, protecting the developmental processes by buffering genetic and environmental perturbations. Similarly, other regulators (e.g., transcription factors and genes involved in translation regulation) may be associated with ASD because their disruption leads to loss of robustness. New examples for regulators that are involved in ASD are topoisomerases. One study found that inhibiting topoisomerase 1 (TOP1) reduces the expression of large number of very long genes, which are particularly expressed in the brain (King et al., 2013). Two other studies provided evidence that Top3β is an RNA topoisomerase that interacts with the fragile X mental retardation protein (FMRP) to regulate the expression of multiple mRNAs that are crucial for neurodevelopment (Stoll et al., 2013; Xu et al., 2013).

**Figure 1:**
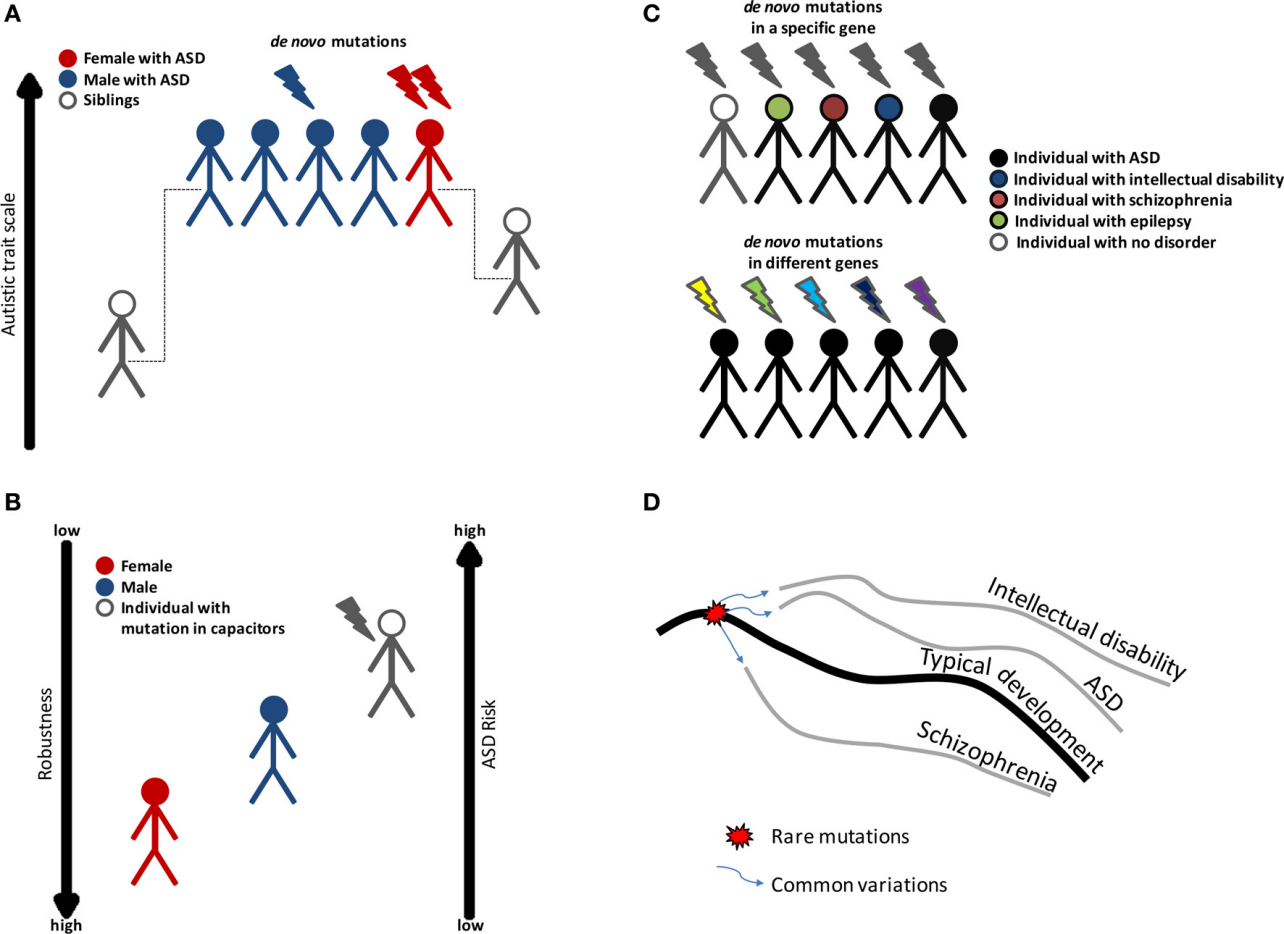
**Variation in ASD risk may be linked to phenotypic robustness. (A)** Evidence to show that females are protected against ASD. ASD is more prevalent in males; however females exhibit a higher rate of genes disrupted by *de novo* mutation. On average, family members of females with ASD have higher autistic trait scores than the family members of males with ASD. **(B)** ASD risk may be associated with phenotypic robustness, and decreased robustness leads to a higher risk of ASD. For this reason, males are more predisposed to ASD than females, and individuals with mutations in phenotypic capacitors have the highest risk. **(C)** ASD is characterized by genetic heterogeneity, incomplete penetrance and variable expressivity. Variations in phenotypic robustness may explain some of the genetic complexity in ASD. **(D)** A model of how rare and common variations cause different neurodevelopmental disorders. A genetic loss of robustness (decanalization) is caused by mutation in regulators that act as capacitors, followed by an abnormal development of the brain. Common variations direct the abnormal development toward a specific trajectory (a specific disorder).

The proposed model is that individuals, in whom the robustness of brain development has been impaired by genetic or environmental insults, will have an increased risk of developing a neurodevelopmental disorder (Figure 1). The specific phenotype will depend on the combination of other genetic and environmental factors including stochastic events. According to this model, individuals with disruptive mutations in regulators, such as chromatin regulators, will have reduced phenotypic robustness (or developmental instability) which may lead to a number of different conditions. In fact, some of the genes found to be disrupted in individuals with ASD were also found in congenital heart disease (Zaidi et al., 2013). Similarly, we propose that being a male is a risk factor for different neurodevelopmental disorders because of reduced phenotypic robustness. Other factors, including the environment, may as well lead to loss of robustness. Furthermore, an individual may carry a mutation that decreases robustness but will have typical development under specific environmental and genetic conditions, which are more stable. Aside from the genetic evidence linking chromatin and other regulators to autism, this model is also attractive as it has the potential to elucidate a large set of puzzling observations in ASD. We highlight some of the evidence for this theory and the evidence for the more classical view in Table 2. We also propose in Table 2 several experiments that could be used to test the loss of robustness theory and its connection to chromatin and other regulators in ASD.

**Table 2:** **Evidence for the classical view and the loss of robustness theory in ASD**.

	**Classical view**	**Evidence for classical view**	**Loss of robustness theory**	**Evidence based on loss of robustness**
Type of genes disrupted in ASD	Genes involved in the formation or function of brain circuits important for social cognition and language	Mutations in genes involved in synaptic function Krumm et al., 2014	Mutations in capacitors^*^	Mutations in chromatin regulators Ben-David and Shifman, 2013; Krumm et al., 2014
Differences in means and in variances of traits	ASD risk is caused by changes in mean values for social traits	Parents from multiplex families show intermediate autism phenotype characteristics Bernier et al., 2012	Increase in variation of cognitive traits in less robust individuals^**^	Males show more variations in cognitive and personality traits than females Hedges and Nowell, 1995; Borkenau et al., 2013
Variable expressivity and incomplete penetrance	The genetic modifier hypothesis: variability in expressivity and incomplete penetrance is due to additional genetic variations	Evidence from multiple hit model Leblond et al., 2012	Individuals with loss of robustness will be at a higher risk for different diseases^***^; Robust individuals will not show a phenotype even in the presence of mutations^****^	Males show higher incidence for all neurodevelopmental disorders (see main text). Individuals with ASD are more likely to have other brain related disorders Simonoff et al., 2008; Memari et al., 2012

Additional experiments and predictions that could be used to provide further evidence for the loss of robustness theory:

* *Perturbation of regulators associated with ASD will cause increase the degree of variation in gene expression*.

** *Animal models with mutations in capacitors linked to ASD will show greater phenotypic variation and increase in fluctuating asymmetry*.

*** *Genetic variants leading to loss of robustness will tend to be shared across diagnostic boundaries, more than other type of variants*.

**** *Animal models carrying mutations in capacitors linked to ASD should have less ability to suppress the effects of other introduced mutations*.

## Reduced robustness in brains of males vs. females

One finding that remains consistent in ASD research is that boys are more likely to be diagnosed with autism than girls. The ratio between males and females is 4:1, but it may increase up to 15:1 for the high functioning end of the spectrum (Wing, 1981). This means that girls are less likely to develop ASD, but when they do, they tend to show a more severe phenotype. There are several explanations for this major difference in prevalence between the sexes. From early prenatal development, through adolescence, males and females have different levels of sex hormones, such as testosterone and estrogen. These hormones are known to have a major influence on brain development, structure and function (Berenbaum and Beltz, 2011). It has been proposed that different hormone levels can increase or decrease the risk of developing ASD (Baron-Cohen et al., 2005).

A different hypothesis that has gained recent support is that there are unknown factors that “protect” females from ASD (Werling and Geschwind, 2013). Recent genome-wide studies showed that on average females with ASD have more *de novo* mutations than males, including CNVs and SNVs (Levy et al., 2011; Iossifov et al., 2012; Neale et al., 2012). For example, in the exome sequencing study of Iossifov et al. they observe, on average, twice the number of *de novo* disruptive mutations in females relative to males with ASD (9 in 29 females [31%] vs. 50 in 314 males [16%]). In addition, *de novo* CNVs in females were larger and included significantly more genes than CNVs in males (Levy et al., 2011). A recent study addressed this hypothesis directly by examining dizygotic twin pairs, comparing autistic traits in siblings of female and male probands that were at the top 90 and 95th percentiles of the distribution of the autistic trait score (Robinson et al., 2013). They showed that siblings of females with ASD had higher scores in autistic traits scale than siblings of males with the disorder. This result, replicated across two nationally-representative samples, implies that in girls a greater load of genetic risk factors is required to cause ASD. Although other studies did not find the same effect (Ozonoff et al., 2011), this was the first study to examine representative community samples without ascertainment bias, and using quantitative measurements that may be more sensitive.

The female protection theory fits with the model of loss of robustness. According to this model, besides the variation between individuals, there is a difference in the average degree of robustness of the brain between males and females. Supporting this suggestion is the higher rate of boys relative to girls in other neurodevelopmental disorders such as attention-deficit–hyperactivity disorder (ADHD), dyslexia, specific language impairment, Tourette Syndrome, and other learning difficulties (Shaywitz et al., 1990; Kadesjo and Gillberg, 2000; Rutter et al., 2003; Simon et al., 2009). There are several possible genetic explanations for the difference in robustness between males and females. The early genetic hypothesis was that some genes involved in ASD are located on the X chromosome (Skuse, 2000). Females inherit two X chromosomes from both parents, while males inherit only one maternal copy. Consequently, females are less vulnerable to mutations in genes on the X-chromosome since they have a second copy that can compensate. The robustness model generalizes this concept of genetic protection in females, by stating that females may have a more robust development of the brain and therefore require larger perturbations to develop ASD and other neurodevelopmental disorders. Robustness may lead to lower prevalence, but it also means that females require a greater genetic insult that may go along with a more severe phenotype.

### Loss of robustness may explain variable expressivity and incomplete penetrance

Genetic findings from recent years show that mutations associated with ASD have low specificity (Figure 1C). Not only are there probably hundreds of genes that may contribute to ASD, many of the genes that were identified until now have also been found to be associated with other neuropsychiatric conditions, including mental retardation, epilepsy, ADHD, and schizophrenia (Betancur, 2011; Devlin and Scherer, 2012; Kirov et al., 2013). This phenomenon is known as variable expressivity, whereby individuals with a certain mutation exhibit differences in phenotypic expression, such as disease severity or symptoms. Variable expressivity among different individuals may arise from individual-specific genetic and environmental factors. It coincides with the finding that around 70% of the individuals with ASD also suffer from other psychiatric conditions (Simonoff et al., 2008; Memari et al., 2012). In fact, the authors cannot state a single example of a gene that causes only ASD when disrupted. Furthermore, some of the rare variants associated with ASD were observed in individuals with normal development, showing that those variants may not be sufficient to cause ASD (Zhao et al., 2007; Ben-Shachar et al., 2009; Beunders et al., 2010; Schaaf et al., 2011; Kirov et al., 2013). The observation that not all people who carry a mutation develop the disease is termed incomplete penetrance. A recent study showed that CNVs associated with ASD or schizophrenia affect cognition in control individuals. The CNVs were considered to have incomplete penetrance; however, the results show that the differences between cases and controls are in fact due to variable expressivity (Stefansson et al., 2014).

One explanation for the variable expressivity and incomplete penetrance is that other genetic variations may modify the phenotype (Schaaf et al., 2011). Evidence for this explanation comes from mouse models of ASD. In mouse models, it is very common to observe a large phenotypic variation dependent on the genetic background. For example, when the *FMR1* (the gene for fragile X syndrome) knockout mouse was crossed with six different inbred strains, only one line (B6.D2 F1 hybrid) showed both social and communication deficits along with repetitive behaviors (Spencer et al., 2011). This means that there are genetic variations between the strains that modify the effect of the mutation without a direct effect on phenotype. In other words, these effects are due to epistatic interactions (Epistasis is when the phenotypic effect of one genotype depends on the genotype of other genes).

In ASD, we suggest to attribute the incomplete penetrance to differences in robustness among individuals. It was demonstrated in Caenorhabditis elegans that incomplete penetrance is a consequence of stochastic variation in gene expression, which is influenced by chromatin regulators and other capacitors (Raj et al., 2010; Burga et al., 2011). Individuals in whom brain development is more robust are expected to show low variation in gene expression and lower penetrance for mutations (Queitsch et al., 2012). Furthermore, in robust individuals some of the genetic variations will be cryptic. In less robust individuals, such as individuals with mutations in phenotypic capacitors, genetic variations that were compensated for will be revealed and contribute to the disease. Different genetic backgrounds, including common and rare variations, could explain the phenotypic variations observed in humans with mutations in the same gene.

## Common and rare genetic variants operate together to increase the risk of ASD

As with many other common complex diseases, one of the main hypotheses about the genetic architecture of ASD was that it is influenced by common variants (Risch and Merikangas, 1996; Risch et al., 1999). In addition to past studies looking at candidate genes, genome-wide association studies were performed in recent years to identify single nucleotide polymorphism (SNPs) associated with ASD (Ma et al., 2009; Wang et al., 2009; Weiss et al., 2009; Anney et al., 2010). While some studies were able to identify SNPs with genome-wide significance (*P* < 5 × 10^−8^), none of those were replicated across studies. Until now, the sample size in GWAS has been modest (487–1558 families), therefore the failure of those studies could not be used as evidence against the contribution of common variants to ASD risk. Although efforts to identify specific SNP associated with ASD have not been very successful, several studies have shown that common SNPs collectively contribute to ASD (Voineagu et al., 2011; Ben-David and Shifman, 2012; Klei et al., 2012), estimated to explain 17–40% of ASD liability (Klei et al., 2012; Lee et al., 2013). The association of rare variants with ASD, on the other hand, has had a long history of success, as described above. But, do common and rare variants operate together? A support for a model that combines rare and common variations in ASD risk comes from a study that showed that both typse of variants affect that same kind of neuronal genes (Ben-David and Shifman, 2012). By dividing the genes in the human genome into functionally related groups, based on pattern of gene co-expression in the human brain, it was shown that genetic risk variants are most enriched in a group of synaptic genes expressed in adulthood, and in another group that contains genes involved in neurogenesis (genes active both during development and adulthood). The “synaptic group” was more enriched with common risk variants, while the “neurogenesis group” with rare variants. Whereas the relative contribution of rare and common variants is now starting to be explored at the population level, we still know very little about how common and rare variations at the individual level act together to cause ASD.

Adaptive robustness can also be achieved through negative epistatic interactions among alleles of common variants (Gibson, 2009). In the presence of risk alleles, selection that reduces the additive genetic effects will result in a more robust phenotype, since the effect of risk alleles is suppressed by other loci. It was suggested that dramatic changes in the environment, such as the ones that accrued in modern times in human urban societies, may result in changes in the epistatic interactions among alleles and reduced robustness (decanalization) (Gibson, 2009). This may explain the increase in incidence for different common diseases, including ASD.

As stated above, loss of robustness may increase the risk of neurodevelopmental disorders, but the specific phenotype may be modified by common genetic variations (Figure 1D). Thus, based on this model, genetic variation associated with ASD could be divided to two types: (1) genetic variation, mainly rare mutations, that reduce the phenotypic robustness of the brain, and (2) genetic variation, such as common variants, that influence brain functions involved specifically with social cognition. These latter variations are cryptic, since they are exposed only in individuals with loss of robustness. The genetic evidence for such a model comes from the low specificity of rare mutations, the disruption of genes that could be considered as master regulators in several diseases, and the high specificity of common variations to ASD. The enrichment of genes involved in transcription regulation was found in ASD, schizophrenia and other neurodevelopmental disorders (Najmabadi et al., 2011; Ben-David and Shifman, 2013; Gulsuner et al., 2013), suggesting that loss of robustness might be a more general cause of neurodevelopmental disorders. Hence, a single rare mutation may be associated with different disorders. In contrast, there is a very modest or non-significant genetic correlation between ASD and other psychiatric disorders, based on polygenic risk scores calculated from common variations (Cross-Disorder Group of the Psychiatric Genomics Consortium, 2013; Lee et al., 2013). While larger GWAS in ASD are needed to validate the low correlation, the current findings suggest that common variations may contribute to the specific phenotypes of ASD in individuals with rare mutations.

## Summary

The classical genetic view of ASD and many other neuropsychiatric disorders is focused on genes that code for neural components that are essential for brain function. In the field of ASD, a great deal of research was focused on genes and proteins that are expressed in the adult brain and function at the synapse, or are regulated by neuronal activity (Zoghbi, 2003). The notion is that ASD is caused by mutations that interfere with brain regions or functions that are involved in the, so called, “social brain.” The recent developments of new techniques, allowing us to examine the autism genome in a genome-wide, unbiased manner, have brought to light another class of genes as associated with ASD risk. These genes are chromatin regulators, which are active during brain development. Based on this finding, and together with the observation of variable expressivity of mutations and the recent evidence for a female protective effect, we propose a unifying framework that connects phenotypic robustness theories with ASD risk (Figure 1). The implication of this theory, if proved true, is that the prevalence of ASD and other neurodevelopmental disorders may be associated with environmental factors that decrease robustness.

### Conflict of interest statement

The authors declare that the research was conducted in the absence of any commercial or financial relationships that could be construed as a potential conflict of interest.
